# The Teaching Digital Competence of Health Sciences Teachers. A Study at Andalusian Universities (Spain)

**DOI:** 10.3390/ijerph18052552

**Published:** 2021-03-04

**Authors:** Julio Cabero-Almenara, Julio Barroso-Osuna, Juan-Jesús Gutiérrez-Castillo, Antonio Palacios-Rodríguez

**Affiliations:** Department of Teaching and Educational Organization, University of Seville, 41013 Seville, Spain; cabero@us.es (J.C.-A.); jbarroso@us.es (J.B.-O.); jjesusgc@us.es (J.-J.G.-C.)

**Keywords:** digital competence, DigCompEdu, higher education, health sciences, teacher training, COVID-19

## Abstract

The impact and benefit that information and communication technologies (ICT) have in the educational field require new teaching skills. This fact has been increased by the recent crisis caused by COVID-19. This study tries to investigate the level of digital teaching competence (DTC) of Higher Education teachers of Health Sciences, and its relationship with several variables. For this, it has the participation of 300 teachers from the 9 universities of Andalusia (Spain). The research is structured through a descriptive (RQ1) and inferential (RQ2) design. The answers given to the DigCompEdu Check-In questionnaire adapted to the Spanish context are analyzed. The results, which show high levels of reliability of the questionnaire (Cronbach and McDonald) and validity (CFA), indicate that the level of competence is basic-intermediate. In addition, the area in which teachers excel is digital resources. For this reason, it is proposed to structure personalized training plans and continue expanding the characteristics of this study at an international level.

## 1. Introduction

If digital teaching competence (DTC) has become necessary at the beginning of the 21st century, what happened with the pandemic produced by COVID-19 has made it more necessary than ever to have teachers who possess a strong command of DTC.

The Council of the EU defines digital competence as that which “involves the safe and critical use of the technologies of the information society for work, leisure and communication” [1]. This presence in society is also perceived in educational institutions, where never before has the teacher had so many technological resources to carry out their professional teaching activities [2].

DTC implies the acquisition of a set of skills, knowledge and attitudes that the teacher must possess for the technical, pedagogical and didactic incorporation of information and communication technologies (ICT) in educational contexts; in short, what a teacher should know for the design, production, use and evaluation for the educational incorporation of ICT in the teaching-learning processes [3,4,5].

Different studies point to the particular benefits of using and combining various technologies and methodologies in medical education [6]. These include experiences with augmented reality [7], 3D printed models [8], simulations with virtual reality [9] and mobile applications [10]. The combination of these technologies enables Health Sciences students to be updated in the dynamic and ever-changing nature of medical education [11].

To analyze the specific competences that digital competence implies, different frameworks have been proposed. Table 1 shows the most consolidated and significant reference frameworks at the international level [12,13,14].

These frameworks seek to identify training needs and propose personalized training itineraries [15]. Along these lines, these frameworks were analyzed in a previous work through the external competence coefficient, where the responses of 179 national and international experts in digital competence were validated [16]. In this study, a comparison of the main competencies and areas of the frameworks is made. The results of the study showed that the DigCompEdu framework was the most valued framework, becoming the most suitable for use in the university context, hence its importance and taking it as the object of study in this research. The study values its pedagogical component very positively. This is one of the main advantages over other frames. On the contrary, the rest of the analyzed frameworks pay special attention to the technological dimension of digital competence; the pedagogical dimension is relegated to the background.

**Figure 1:**
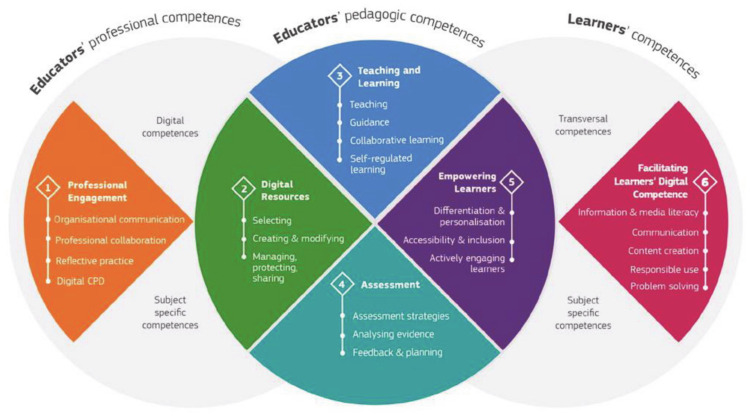
Conceptual view of the DigCompEdu framework [12].

DigCompEdu framework focuses, as shown in Figure 1, on three large competence dimensions: professional, pedagogical and student competences. The framework aims to support national, regional and local efforts to promote the digital competence of educators, offering a European reference space with a common language and logic.

The Professional Engagement dimension focuses on the teachers’ work environment in order to be able to interact with the different agents of the educational community (A); the Digital Resources dimension is related to the creation and distribution of digital resources in the classroom, respecting copyright norms (B); the Teaching and Learning dimension is related to knowing how to design and plan the use of technology in student learning, with active methodologies focused on this group (C); the Assessment dimension focuses on the use of ICT resources for the evaluation of students (D); the dimension Empowering Learners is related to ensuring digital access to all students, offering learning activities adapted to their level of competence, as well as their interests and educational needs (E); and finally, Facilitating Learners´ Digital Competence (F).

At the university level, research shows a lack of teacher training for the incorporation of ICT in teaching [5,17] and the need to establish training plans. Regarding digital competences in Health Sciences, digital competence was analyzed in the Faculty of Health Sciences of the UNACH (Ecuador) [18], finding a deficiency in the lack of digital training related to management and generation of information, as well as the dissemination of knowledge. Other studies [19,20], however, have shown the special interest of health professionals in knowing and acquiring the digital competence that society demands in the 21st century.

Stud and analysis to establish training actions is more necessary in the pedagogical-didactic component than in the technological-instrumental [5]. This indicates that the analysis of this competence is necessary, because its low dominance has repercussions in a lower and unqualified educational use of ICT by the teacher [21]. On the other hand, as its mastery has a transversal impact on other competences that the teacher must have, its study and analysis, it becomes more necessary [22].

For this reason, different authors defend that teaching models should be approached from different perspectives that lead them to focus more on the pedagogical than on the didactic [23]. Pedagogical training is presented as a good predictor for the didactic use of ICT [24] and, at the same time, it focuses on improving their beliefs and attitudes towards the educational use of ICT [25]. These aspects will facilitate the educational incorporation of ICT and the performance of innovative practices with them.

This study arises from the educational problems that emerged from the COVID-19 crisis, where the digital skills of teachers have been called into question. Consequently, this study tries to answer the following research questions:

1. What are the digital teaching competences of the Andalusian university teachers of Health Sciences? (RQ1)
2. Are there significant differences based on different established variables? (RQ2)

## 2. Materials and Methods

An ex-post facto investigation is proposed, since the nature of the variables is not modified. In this case, a cross-sectional design is carried out since a single measurement is carried out in a certain context (teaching during the crisis caused by COVID-19). The same methodology has been used in studies related to this research [11,18,19].

The research is structured through a descriptive (RQ1) and inferential (RQ2) design. In the same way, data on the reliability and validity of the instrument are also provided.

### 2.1. Objectives

To answer the research questions, the objectives were threefold: O1) To know the reliability index of the diagnostic instrument, O2) analyze the domain in DTC shown by university teachers of Health Sciences during the pandemic produced by COVID-19, and O3) analyze if there are significant differences in the DTC shown by Health Sciences teachers according to the variables: gender (3.1), age (3.2), teaching experience (3.3), years they have been using ICT (3.4) and the technological domain expressed (3.5).

### 2.2. Sample

The sample has been selected by intentional sampling and convenience. 300 university teachers who teach degrees in Health Sciences at the 9 Andalusian public universities (Spain) participated in this study. Table 2 shows the participation index by Andalusian universities (Spain).

There was a participation of 158 women and 142 men. The majority were over 40 years old (78%), and stated that they had more than 10 years of teaching experience (68.7%). Regarding the time they have been using ICT as a learning tool, more than half stated that they have been integrating them in the classroom for 10 or more years (55.3%). Also, the vast majority showed a positive or very positive attitude when asked about the level of curiosity they have regarding ICT (87.4%). In the same way, almost all respondents claimed to be a user of between 2 and 4 social networks (67.3%).

Together, the vast majority claimed to have a high or very high technological proficiency in terms of using computers (96.6%), tablets (90.7%), smartphones (93.3%) and the internet (84.3%).

### 2.3. Instrument

DTC was measured with the DigCompEdu Check-In instrument, adapted to the Spanish context [4]. The instrument is made up of 6 competence dimensions and a total of 22 items (Figure 1):

1. Professional engagement: focused on the work environment of teachers, where their ability to use digital technologies to improve teaching and interact professionally with colleagues, students, family and different agents of the educational community is expressed. This area of competence includes 4 items: organizational communication, professional collaboration, reflective practice and digital training.
2. Digital resources: related to the sources, creation and distribution of digital resources. One of the key competencies that any teacher must develop is to identify good educational resources. Additionally, she must be able to modify, create and share them to suit her goals, learners and teaching style. At the same time, she must know how to use and manage digital content responsibly, respecting copyright rules and protecting personal data. This includes 3 items: selection; creation and modification; and administration, exchange and protection.
3. Teaching and learning: knowing how to design, plan and implement the use of digital technologies in the different stages of the teaching and learning process. In addition, a change in approaches and methodologies that are student-centered is advocated. This encompasses 4 items: teaching, guidance, collaborative learning and self-directed learning.
4. Assessment: linked to the use of digital tools and strategies in the evaluation and improvement of teaching-learning processes. Digital technologies can improve existing assessment strategies and lead to new and better assessment methods. Space in this area of competence is given to 3 items: evaluation strategies, analysis of evidence and tests, and feedback and planning.
5. Empowering learners: one of the key strengths of digital technologies in education is their potential to promote the active participation of students in the learning process and their autonomy over it. In addition, digital technologies can be used to offer learning activities tailored to each student’s level of competence, interests and learning needs. This area of competence is composed of 3 items: accessibility and inclusion, differentiation and personalization, and active participation of the students.
6. Facilitating learner´s digital competence: how to develop and facilitate the students’ digital competence, through 5 items: information and media literacy, digital communication and collaboration, creation of digital content, responsible use and well-being, and digital problem solving.

Each item is measured on a 5-interval Likert scale. In each of them, the participants indicate to what extent they reflect their own teaching practice by selecting one of the five options. These are organized progressively, reflecting the general progression logic of DigCompEdu (competence levels) through an internal scoring system. This progression follows the structure of: no commitment (0 points), partial knowledge (1 point), occasional use (2 points), increasing use (3 points) and systematic and comprehensive use (4 points).

The anonymous questionnaire was sent through the online platform EuSurvey and it can be consulted through the following link: https://bit.ly/2ZyfyQR.

### 2.4. Data analysis Procedure

Consistent with the research questions, data analysis includes several procedures. First, the internal structure (reliability) of the instrument is verified through the Cronbach’s Alpha and McDonald’s Omega, as well as the construct validity with the confirmatory factor analysis (CFA), convergent validity and discriminant validity. As statistical software, SPSS V.26 and AMOS V.24 are used to check the modeling of structural equations (SEM) on the relationships between the items of the instrument. After that, a descriptive study of the data is made using statistics of central tendency (mean) and dispersion (standard deviation). To calculate the total competence level and competence by dimension, a summation of the related items over 4 points is made [4].

For the contrast of the different hypotheses, non-parametric contrast statistics were applied: U of Mann–Whitney and K of Kruskal–Wallis with a post-hoc test (Dunn’s test). 

## 3. Results

### 3.1. Reliability and Validity (O1)

The first objective of our work was to know the reliability index of the instrument used for diagnosis. To check the validity of the construct, a series of indices, together with the values necessary for the model proposed in the CFA to be satisfactory [26], is proposed. Mardia´s coefficient (CM) shows multivariate normality by finding values between 3 and 70 [27]; residual mean square root (RMR) values lower than 0.10 are considered favorable [28]; a Chi-square ratio with degrees of freedom (χ2/gl; CMIN/DF) values lower than 5 indicate a good fit [29]; the non-normed fit index (NNFI), the Tucker–Lewis index (TLI), the comparative fit index (CFI) and the incremental fit index (IFI) consider values above 0.90 as a good fit. Mean square error of approximation (RMSEA) values between 0.05 and 0.08 [30], composite reliability (CR) coefficients with values greater than 0.7, average variance extracted (AVE) with values greater than 0.5 [31], and maximum shared variance (MSV) with a value less than the AVE coefficient [32] are all considered good. Table 3 shows the coefficients for each of these adjustments.

For the reliability analysis, the Cronbach’s Alpha and McDonald’s Omega coefficients reveal very satisfactory levels in the total of the instrument, as well as satisfactory results in the different dimensions. 

### 3.2. Teaching Digital Competence Level (O2)

Having corroborated the results obtained in terms of validity and reliability of the instrument used, our second objective is to analyze the domain in DTC that these teachers show in their teaching during the pandemic produced by COVID-19. Table 4 represents the mean values obtained for each item of the instrument (out of 4 points), as well as their standard deviation.

Values range from 1.18 (basic level) to 2.33 (intermediate level). Specifically, teachers show problems (basic level) in teaching students how to behave safely and responsibly online, using digital technologies to offer students personalized learning opportunities and analyzing all available data to identify students who need additional support. The competences that stand out (intermediate level) are to systematically use different digital channels to improve communication, create your own digital resources and modify existing ones to adapt them to my needs as a teacher, and use digital technologies to acquire and document knowledge when students work in teams. 

**Figure 2:**
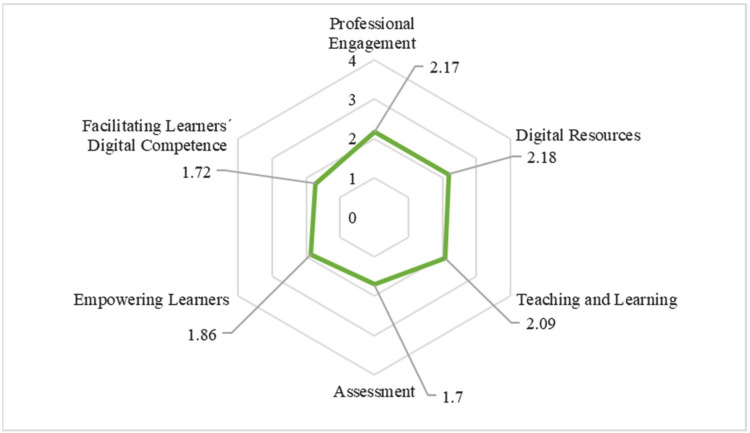
Average value of the competence level by DigCompEdu areas.

At a general level, the mean scores obtained in each dimension are represented in Figure 2. These values have been calculated from the items (Table 3) that make up each dimension.

The average value reached in the instrument as a whole is 1.95 points with a standard deviation of 0.69. This leads us to point out that, in general, the level of competence is basic-intermediate. In addition, ordering from lowest to highest, the results by dimensions are as follows: Assessment (1.7); Facilitating Learners´ Digital Competence (1.72); Empowering Learners (1.86); Teaching and Learning (2.09); Professional Engagement (2.17); Digital Resources (2.18). Therefore, in general, teachers present basic levels of DTC, although they are considered moderately competent in professional commitment and digital resources. It is significant that the highest score was in digital resources dimension.

### 3.3. Contrast Analysis (O3)

The third objective of this research is to analyze whether there are significant differences in the DTC shown by Health Sciences teachers based on the variables: gender (O3.1), age (O3.2), teaching experience (O3.3), years of using ICT (O3.4) and the expressed technological domain (O3.5).

Regarding the gender variable, the results achieved in the Mann–Whitney U test are 10146000 with a significance of 0.153. These values allow accepting the H0 formulated in reference to significant differences based on gender. Consequently, it can be stated that there are no significant differences in the dominance of DTCs based on gender (O3.1).

For the analysis of the significance of age, experience and time of use, the Kruscal–Wallis test was applied, obtaining the results that are presented in Table 5.

The values reached allow the rejection of all H0 at a significance level of *p* ≤ 0.05. There are statistically significant differences between the level of DTC and the age, experience and time of ICT use in the classroom.

Regarding its technological domain, Table 6 presents the Mann–Whitney U value.

In this case, H0 is also rejected at a significance level of *sig.* ≤ 0.01. There are statistically significant differences between the level of DTC and the technological domain.

Table 7 shows the values that are statistically significant (*sig.* ≤ 0.05). They are drawn from the application of Dunn’s multiple comparisons test in the Kruskal–Wallis test for the rest of the variables under study.

In reference to age (O3.2), fundamental differences have been established between teachers of “50–59 years” and those of “30–39 years”, the second being the group with the highest level of DTC. Additionally, between the teachers of “50–59 years” and those of “40–49 years”, in this case also the second ones are the ones with the highest level of DTC. Therefore, it can be stated that the null hypothesis is rejected for the two cases mentioned above.

Regarding the years of experience (O3.3), there are significant differences between teachers with seniority of “1–3 years” and those of “10–14 years”, with the latter having greater digital competence; between those with “1–3 years” of experience and those with “4–5 years”, the latter having higher DTC; between those of “20 or more years” and those of “10–14 years”, with the latter having a higher DTC; and, finally, between those “20 or more years” and those with “4–5 years”, with the second having a higher DTC.

Regarding the time that the surveyed teachers have been using ICT (O3.4), the fundamental differences are:

- Between teachers who “do not use technology as an educational tool”, those who have been using it for “less than a year”, “1–3 years”, “4–5 years”, “6–9 years”, “10–14 years”, “15–19 years” and “20 years or more”, there was, as expected, a higher DTC in the latter.
- Between teachers who have been using ICT for “less than 1 year” and those who have used it for “4–5 years”, there was a higher DTC in the latter.
- Between teachers who have been using ICT for “15–19 years” and those who have been using them for “10–14 years”, there was a higher DTC in the latter.
- Between teachers who have been using ICT for “1–3 years” and those who have used them for “4–5 years”, there was a higher DTC the latter.

Regarding the technological domain (O3.5), there are significant differences between the groups of teachers with “low” skill in the use of technologies and those with “very low” skill, with the teachers included in the second group presenting a higher DTC; between those with “very high” skill and those with “very low” skill, the teachers included in the first group having a higher DTC; and between those with “high” skill and those with “very low” skill, the teachers included in the second group having a higher DTC.

## 4. Discussion and Conclusions

For the presentation of the conclusions, the same premise established in the presentation of the results is followed. Therefore, a response is given to each of the objectives raised at the beginning of this study.

The results of the different analyzes carried out corroborate that the DigCompEdu Check-In instrument, adapted to the Spanish context [4] and used with professors from the Health Sciences branch during COVID-19, is presented as a valid and reliable instrument to measure DTC (O1). Consequently, its use becomes feasible given its psychometric properties. Likewise, it must be taken into account that it is presented as a self-perception of reality, and not as reality itself.

Regarding the second objective of the research, it can be concluded that the results obtained present an overview of the teaching profile of Health Sciences in higher education, in reference to professionals’ DTC, in a specific period during the pandemic caused by COVID-19; professionals with, for the most part, more than 10 years of experience and high technological mastery. These results are similar with that of another study carried out [18], which places the technical level of medical teachers above the pedagogical level. At the same time, it has been shown that the teachers surveyed obtain scores that place them at basic-intermediate levels of DTC [33]. Specifically, they stand out in the area of digital resources, which may be related to the growing interest in knowing and using new resources in the classroom as a response to the demand for teaching innovation with ICT [19,20,34].

In relation to these results, and taking into account the measurement period of the same, it is indicated that COVID-19 has brought different changes and tensions in the educational system as a result of several factors, and has brought a rapid and strong transition towards training at distance eminently supported by technology [19,35]. In this context, weaknesses have emerged derived from teacher training, the lack of technology, the lack of educational resources to be used in distance virtual training and the lack of credibility regarding the effectiveness of this training modality [18,20,36]. Therefore, the results found the need to carry out teacher training plans where special interest is directed to the educational use of ICT and technologically empowering students, an aspect in which it is completely necessary to train medical students [18].

It is understood that the conclusions proposed in this study should be interpreted with caution. The type of non-experimental design and the size of the sample imply some restrictions for the generalization and application of the results. Future research could consider larger and differentiated samples by type of knowledge area. Furthermore, it would be important to implement international studies in order to extend the scope of the results and statistical techniques. Therefore, the purpose is to continue improving and expanding the characteristics of this study, in order to validate these preliminary findings.

The present study also aims to know if there are significant differences between variables such as gender, age, experience, years of use of technologies, time spent on technologies and mastery of technologies and level of DTC shown by teachers in the Health Sciences branch of all Andalusian universities (O3).

In the last decade, there are many studies that have focused on analyzing the digital divide of university teaching staff based on the gender variable in terms of their DTC. These investigations highlight that the mean scores obtained by women are higher than those of men [5,37]. Also, along another line, there are studies that place men at higher levels of DTC [38,39]. Taking into account the scores obtained in this study, it is indicated that there are no significant differences between gender and the DTC of teachers in the branch of Health Sciences of Andalusian public universities. Therefore, it is concluded that gender is not a significant factor/variable for DTC. These results are consistent with other previous investigations [40].

Some studies have shown that age is not a significant factor/variable for the digital competence of teachers [41]. Based on the results obtained, it is clear that this variable is significant, since the teachers analyzed between 30 and 49 years of age show a higher DTC. This statement is consistent with other studies that indicate that younger teachers demonstrate higher levels of competence than older teachers [42,43]. It can be said that the group of teachers aged in this range have a positive attitude towards the use of ICT and a greater interest in their training in these competencies. This fact may be related, as suggested by other related investigations [44], to the fact that, in the initial training plans of these teachers, the development of digital competence was more visible and latent. Similarly, it is pointed out that they have had access to digital technologies before their older colleagues, a fact that has enabled them to integrate them into their teaching profession.

Related to the age variable and to the studies mentioned above, through the results obtained in terms of years of experience, it can be affirmed that teachers with experience between 4 and 14 years old have a higher level of DTC than their peers, newer classmates and more veteran ones, coinciding with the data obtained in the previous variable. This aspect may be due to the use they make of technologies. In the case of young people, it may seem that they have greater digital competence, but making a more superficial use of these, since they are in the initial phase of professional development. Therefore, it would be necessary for them to develop pedagogical competencies for the use of technologies in an educational context, a theoretical model that underlies DigCompEdu compared to the use of other competency frameworks. At the other extreme, teachers with more experience, and therefore older, tend to make a more conservative use of technology. However, what has been said does not mean that they are not critical or do not show better judgment regarding the use of technology with their students [44].

Regarding experience in the use of ICT, there are significant differences between teachers who do not use technologies and the rest, showing that there are significant differences between teachers with experience in the use of ICT with respect to the domain that these teachers present from their DTC. In this case, teachers who have some experience in the use of ICT show a higher level of DTC, as is the case with teachers who rate their skills with technologies as “high” and “very high”.

As a future line of research, it is advisable to extend the study to other communities and contexts. In this way, it would be possible to have access to the data of professors in the Health Sciences branch of different universities and to establish personalized training itineraries.

## Figures and Tables

**Table 1:** More consolidated and significant competence frameworks.

Framework	Institution	Competence Domains
European Framework for Digital Competence for Educators-DigCompEdu	Joint Research Center	6 competence areas: Professional engagement Digital resources Teaching and learning Assessment Empowering learners Facilitating learners’ digital competence
ISTE Standards	International Society for Technology in Education	7 roles or profiles that a teacher can go through in their professional development and which involve different actions to be undertaken by the teacher, these roles are: Apprentices Leaders Citizens Collaborators Designers Facilitators Analysts
Common Framework for Teaching Digital Competence	Ministry of Education of Spain	5 areas of competence: Information and information literacy Communication and collaboration Creation of digital content Security Problem resolution
UNESCO Competency Framework ICT for Teachers	UNESCO	Teachers must acquire competencies to: Understand ICT in educational policy Curriculum and assessment Pedagogy Application of digital skills Organization and administration Professional
Digital teaching framework	Ministry of Education UK	Set for 7 key areas: Planning Approach Employability of the Student Specific Teaching Evaluation Accessibility and Inclusion Self
ICT skills and standards for the teaching profession	Ministry of Education of Chile	5 dimensions: Technique Pedagogical Management Development and Responsibility Social, Ethical and Legal
ICT skills for teacher professional development	Ministry of Education of Colombia	5 competences that teachers must possess to have an effective use of ICT: Technological Communicative Pedagogical Management Research

**Table 2:** Participation index by Andalusian universities (Spain).

University	N	%
Sevilla	62	20.67
Pablo de Olavide	27	9.00
Granada	52	17.33
Huelva	22	7.33
Cádiz	36	12.00
Córdoba	32	10.67
Málaga	30	10.00
Jaén	16	5.33
Almería	23	7.67
Total	300	100.00

**Table 3:** Construct, discriminant and convergent validity and reliability of the instrument.

	C.M.	CFI	TLI	IFI	NNFI	RMR	RMSEA	90% CI
AFC	22.495	0.946	0.931	0.942	0.931	0.0344	0.058	0.055–0.065
Dimensions		A	B	C	D	E	F	TOTAL
CR		0.731	0.721	0.802	0.820	0.790	0.780	-
AVE		0.584	0.525	0.603	0.614	0.554	0.540	-
MSV		0.872	0.871	0.820	0.821	0.790	0.787	-
Alpha		0.747	0.701	0.742	0.821	0.808	0.831	0.940
Omega		0.953	0.955	0.953	0.951	0.953	0.965	0.991

**Table 4:** Mean and standard deviation of DigCompEdu items.

Item	M	SD
I systematically use different digital channels to improve communication with students, families and my colleagues. For example: emails, WhatsApp type messaging applications, blogs, the school website…	2.29	0.718
I use digital technologies to work with my colleagues inside and outside my educational organization.	2.16	0.897
I actively develop my teaching digital competence.	2.16	1.086
I participate in online training courses. For example: online administration courses, MOOCs, webinars…	2.5	1.178
I use different internet sites (web pages) and search strategies to find and select a wide range of digital resources.	2.19	1.004
I use my own digital resources and modify existing ones to adapt them to my needs as a teacher.	2.32	0.928
I protect sensitive content safely. For example: exams, grades, personal data…	2.05	1.105
I carefully consider how, when and why to use digital technologies in class, to ensure that their added value is used.	2.03	1.106
I monitor my students’ activities and interactions in the online collaborative environments we use.	2.08	1.165
When my students work in groups or teams, they use digital technologies to acquire and document knowledge.	2.33	1.159
I use digital technologies to allow students to plan, document, and assess their learning on their own. For example: self-assessment tests, digital portfolio, blogs, forums…	1.91	1.066
I use digital assessment strategies to monitor students’ progress.	1.75	0.946
I analyze all available data to identify the students who need additional support.	1.58	0.913
I use digital technologies to provide effective feedback.	1.78	0.809
When I propose digital tasks, I consider and address possible problems such as equal access to digital devices and resources; compatibility problems or low level of digital competence of students.	2.02	1.336
I use digital technologies to offer students personalized learning opportunities. For example: assigning different digital tasks to address individual learning needs, taking into account preferences and interests…	1.42	1.330
I use digital technologies so that students actively participate in class.	2.15	0.886
I teach students how to evaluate the reliability of information searched online and to identify erroneous and/or biased information.	1.87	0.977
I propose tasks that require students to use digital media to communicate and collaborate with each other or with an external audience.	1.81	0.899
I propose tasks that require students to create digital content. For example: videos, audios, photos, presentations, blogs, wikis…	1.93	1.116
I teach students how to behave safely and responsibly online.	1.18	0.940
I encourage students to use digital technologies creatively to solve specific problems. For example, overcoming obstacles or emerging challenges in their learning process.	1.81	0.945

Note: The scale of values is between 0 and 4 points, where the values between 0 and 1 represent a low level of competence, 2 and 3 points an intermediate level and 4 a high level.

**Table 5:** Kruskal–Wallis tests: age, experience and use time.

Variable	Kruskal–Wallis	df	Sig.
Age	22,878	5	0.037
Experience	15,735	5	0.008
Time of use of ICT	26,686	7	0.000

**Table 6:** Relationship of the technological domain with digital teaching competence (DTC).

Variable	Mann–Whitney U	W	Z	Sig.
Technological domain	2974.00	12,019	−5.007	0.00

**Table 7:** Significant results between the variables under study and the DTC shown by the Health Sciences teachers.

Variable	Contrast Groups	Test	Dev. Error	Dev. Test	Sig.
Age	50–59 years–40–49 years	32.93	13.47	2.44	0.02
	50–59 years–30–39 years	42.09	15.09	2.79	0.01
Experience	1–3 years–10–14 years	−46.40	18.75	−2.48	0.01
	1–3 years–05.04 years	−54.78	20.94	−2.62	0.01
	20 or more–10–14	40.55	14.91	2.72	0.01
	20 or more–4–5 years	48.94	17.59	2.78	0.01
Timeuse ICT	notuse–Less than 1 year	−112.50	53.10	−2.12	0.03
	No use–15–19 years	−123.48	44.93	−2.75	0.01
	No use–1–3 years	−130.44	45.06	−2.90	0.00
	No use–6–9 years	−155.46	44.99	−3.46	0.00
	No use–20 or more	−155.57	45.20	−3.44	0.00
	No use–10–14 years	−160.88	44.65	−3.60	0.00
	No use–4–5 years	−192.60	47.49	−4.06	0.00
	15–19 years–10–14 years	37.40	15.91	2.35	0.02
	15–19 years–4–5 years	69.12	22.70	3.05	0.00
	1–3 years–4–5 years	−62.16	22.94	−2.71	0.01
Technological domain	Low–Very low	−176.06	61.70	−2.85	0.00
	Very high–Very low	−119.06	36.06	−3.30	0.00
	High–Very low	−112.06	43.89	−2.55	0.01
